# Oleoylethanolamide Alleviates Hepatic Ischemia-Reperfusion Injury via Inhibiting Endoplasmic Reticulum Stress-Associated Apoptosis

**DOI:** 10.1155/2022/2212996

**Published:** 2022-03-21

**Authors:** Shunli Qi, Qi Yan, Zhen Wang, Deng Liu, Mengting Zhan, Jian Du, Lijian Chen

**Affiliations:** ^1^Department of Anesthesiology, The First Affiliated Hospital of Anhui Medical University, Hefei 230032, China; ^2^Department of Anesthesiology, Tongling People's Hospital, Tongling 244000, China; ^3^School of Basic Medical Sciences, Anhui Medical University, Hefei 230032, China; ^4^Key Laboratory of Anesthesiology and Perioperative Medicine of Anhui Higher Education Institutes, Anhui Medical University, Hefei 230032, China; ^5^Department of Biochemistry and Molecular Biology, School of Basic Medical Sciences, Anhui Medical University, Hefei 230032, China; ^6^Infectious Disease Research Center, Anhui Medical University, Hefei 230032, China

## Abstract

Liver ischemia/reperfusion (I/R) injury is a primary complication in major liver surgery. Our previous study about proteome profiling has revealed that the PPAR signaling cascade was significantly upregulated during liver ischemia/reperfusion. To elucidate the potential mechanisms of PPAR*α* involved in I/R injury, we used oleoylethanolamide (OEA), the peroxisome proliferator-activated receptor alpha (PPAR*α*) agonist, in this study. We demonstrated a protective role of OEA on liver I/R injury by using a mouse model of partial warm ischemia-reperfusion and hypoxia-reoxygenation model of hepatocytes. These effects were caused by ameliorating liver damage, decreasing the level of serum ALT and AST, and reducing the apoptosis of hepatocytes. Furthermore, a mechanistic study revealed that OEA regulated endoplasmic reticulum (ER) stress by activating PPAR*α*, thereby reducing ER stress-associated apoptosis to attenuate liver I/R injury. Briefly, these data first proposed that OEA-mediated PPAR*α* activation could be an effective therapy against hepatic ischemia/reperfusion injury.

## 1. Introduction

Liver ischemia-reperfusion (I/R) injury is a common pathological process during varieties of clinical conditions, including shock, trauma, transplantation, and liver resection [1]. Liver I/R injury is complex and associated with anaerobic metabolism, Ca^2+^ overload, damage of mitochondrial structure and function, and oxidative stress reaction [2]. It is well known that hepatocytes are the main cell type in the liver, which produced huge amounts of proteins. Considering large requirement for protein synthesis, hepatocytes contain abundant endoplasmic reticulum (ER) structures and are susceptible to ER perturbation and ER stress. An imbalance of endoplasmic reticulum milieu can cause ER stress, which leads to the activation of the unfolded protein response (UPR). UPR is a defense mechanism against misfolded proteins. It is regulated by ATF6, PERK, and IRE-1, while the three ER membrane sensors became inactive by binding to Grp78. The UPR constitutes a highly conserved and intricately regulatory pathway that maintain ER homeostasis. When ER stress is prolonged or excessive, UPR promotes apoptotic cell death via different brokers, including C/EBP homologous protein (CHOP) and Caspase12. However, in liver I/R injury, excessive hypoxia, ischemia, oxidative stress, and other factors may aggravate the ER stress response [3]. Therefore, inhibition of excessive ER stress may provide a potential therapy for hepatic I/R injury.

Peroxisome proliferation-activated receptor (PPAR) is a nuclear hormone receptor, that is, a ligand-dependent intracellular protein. There are three subtypes of PPAR, including PPAR*α*, PPAR*β*/*δ*, and PPAR*γ* [4]. PPAR*α* is highly expressed in a variety of tissues including liver and plays an important role in fatty acid oxidation [5]. PPAR usually heterodimerizes with 9-cis-retinoic acid receptor (RXR) to form a complex and then acts on the promoter region of the target gene. When activated by agonist, the heterodimeric complex recruited transcriptional coactivators and regulated gene transcription to control lipid and carbohydrate metabolism [6]. Oleoylethanolamide (OEA) is an endogenous lipid mediator, derived from the monounsaturated fatty acid. It is well known for a variety of biological functions, including lipid metabolism, scavenging free radicals, and anti-inflammatory processes [7]. Many positive effects of OEA are believed to depend on the activation of PPAR*α* [8]. Our previous study has revealed that PPAR signaling cascade was significantly upregulated during liver ischemia/reperfusion. Therefore, it is valuable to investigate the effect of OEA on hepatic I/R. Herein, we report a protection of OEA on liver I/R injury, embodied in ameliorating liver damage, decreasing the level of ALT and AST, and reducing the apoptosis of hepatocytes. Therefore, we further investigated the underlying mechanism of OEA in preventing liver I/R injury.

## 2. Materials and Methods

### 2.1. Materials and Reagents

OEA, DMSO, PEG300, and Tween-80 were purchased from Sigma Chemical (USA). Tunicamycin (TM) was purchased from MCE (NJ, USA). Dulbecco's modified Eagle's medium (DMEM) and fetal bovine serum (FBS) were purchased from Viva Cell Biosciences (China). RIPA Lysis Buffer, PMSF, Cell Counting Kit-8 (C0037), DAB Horseradish Peroxidase Color Development Kit, lactate dehydrogenase (LDH), Cytotoxicity Assay Kit, and TUNEL Apoptosis Assay Kit (C1086) were purchased from Beyotime (China). PPAR*α* antibody (Cat: 15540-1-AP), Grp78 antibody (Cat: 11587-1-AP), CHOP antibody (Cat: 15204-1-AP), Caspase 12 antibody (Cat: 55238-1-AP), cleaved-Caspase 3 (Cat: 19677-1-AP), Bcl2 (Cat: 12789-1-AP) antibody, Bax antibody (Cat: 50599-2-Ig), *β*-actin antibody (Cat: 66009-1-Ig), and HRP-conjugated goat anti-mouse/rabbit IgG (SA00001-1/2) were purchased from Proteintech (China). ALT assay kit (C009-2-1) and AST assay kit (C010-2-1) were purchased from Jiancheng Bioengineering Institute (Nanjing, China). Annexin V PE Apoptosis Detection Kit (559763) was purchased from BD Pharmingen (NJ, USA). Lipofectamine™3000 and TRIzol were purchased from Invitrogen. SYBR Green® Premix was purchased from TaKaRa. Chemiluminescent kit was purchased from NCM Biotech (Suzhou, China).

### 2.2. Animal

Male *BALB/c* mice (6-8 weeks, 23–27 g) were purchased from the Anhui Experimental Animal Center (Hefei, China). These mice were housed in isolator cages with free access to food and water in an environment with standard temperature (20-25°C), humidity (40-60%), and lighting conditions (12 h light/dark cycle). All experiments were approved by the Animal Care and Use Committee of Anhui Medical University (20190214, LLSC2019022).

### 2.3. Mouse Warm Liver I/R Injury Model and Experimental Design

The mice were briefly anesthetized with pentobarbital, and the blood supply of left and middle liver lobes (about 70%) was blocked by using an atraumatic clip. After 1 h of liver ischemia, the atraumatic clamp was removed gently for reperfusion, and the mice were euthanized after 6 h of reperfusion [3, 9–11]. Liver tissue and serum were collected immediately for subsequent analysis. The sham operation group underwent the same operation except for vascular occlusion. OEA (20 mg/kg BW) dissolved in a solution (10% DMSO+40% PEG300+5% Tween-80+45% saline) was intraperitoneally injected 1 hour before liver ischemia surgery. The dosage of OEA is determined on the basis of preliminary research. TM (1 mg/kg BW) dissolved in a same solution was intraperitoneally injected 24 h before hepatic ischemia, the dose and route of administration of TM according to previous reports [12]. Inject an equal volume of solution as a carrier control. The mice were assigned to six groups: (1) sham, (2) I/R, (3) vehicle-I/R, (4) OEA-I/R, (5) TM-I/R, and (6) TM-OEA-I/R. All experiments were repeated at least three times.

### 2.4. Analysis of Serum Samples

The expression level of serum ALT and AST, which is an indicator of liver injury, was measured by commercial kit according to the manufacturer's protocol.

### 2.5. Histopathology

The liver tissue was fixed with 4% paraformaldehyde for 48 hours, embedded in paraffin, and cut into 5 *μ*m thick sections. The liver tissue was stained with hematoxylin and eosin (HE), and the necrotic area was analyzed with ImageJ V1.8.0 software. The percentage of the necrotic area of each mouse to the total area was blindly quantified over 5 fields of view [13].

### 2.6. Immunohistochemistry

5.0 *μ*m thick liver sections were prepared from paraffin-embedded tissues, deparaffinized, and rehydrated. After antigen retrieval, using 3% hydrogen peroxide, the intrinsic peroxidase activity was blocked and 3% BSA blocked a specific antibody-binding sites. The sections were incubated with specific primary antibody at 4°C for 12 h. After washing, the sections were incubated with secondary antibodies for 1 h at about 20°C, and the immunoreactive cells were visualized using DAB. Stained sections were observed under microscope.

### 2.7. TUNEL Staining

To detect cell apoptosis induced by ischemia-reperfusion, TUNEL was performed using the TUNEL apoptosis assay kit according to the manufacturer's protocol. The TUNEL-positive cells were quantified using ImageJ V1.8.0 software.

### 2.8. Cell Culture

The HepG2, human hepatocellular carcinoma cell line, was purchased from the American Type Culture Collection (ATCC, USA). The cells were cultured in DMEM supplemented with 10% FBS, and the cells were maintained in a humidified atmosphere with 5% CO_2_.

### 2.9. Oxygen-Glucose Deprivation and Reoxygenation (OGD/R) and Drug Administration

To mimic ischemia-reperfusion injury *in vitro*, OGD/R model was performed according to previously described [14]. The HepG2 cells were cultured in serum/glucose-free DMEM under a humidified atmosphere of 5% CO_2_ and 95% N_2_ for 1 h and then returned to normal medium and conditions. TM (1 *μ*g/ml, dissolved in DMSO) was added to the medium for 6 h before OGD, and OEA (10 *μ*M, dissolved in DMSO) was added before 1 h. The dose of TM was determined based on the previous research [15], and the dose of OEA was determined based on a pilot study. DMSO was used as vehicle control.

### 2.10. Cell Viability and Cytotoxicity Assay

Cell viability was determined by Cell Counting Kit-8 assay according to the manufacturer's instructions. The cells were cultured in 96-well plate. At the end of treatment, the CCK-8 reagent was added and incubated at 37°C for 1 h. The cell viability was determined by measuring the absorbance at 450 nm, and cytotoxicity was determined by LDH release. After treatment, the supernatant was collected immediately to determine the LDH activity by measuring the absorbance at 490 nm.

### 2.11. Flow Cytometry

For apoptosis analysis in the HepG2 cell, Annexin V-PE/7-AAD kit was used to quantify apoptotic cells according to the instructions and then incubated in 1 × binding buffer containing 7-AAD and PE Annexin V for 15 min. Flow cytometry was detected within 1 h after staining, and the cells stained positive for PE Annexin V were selected for apoptosis.

### 2.12. Western Blotting

Cells or liver tissues were prepared by homogenization in RIPA lysis buffer containing 1% phenylmethylsulfonyl fluoride (PMSF). The lysates were electrophoresed in SDS-PAGE and then transferred to nitrocellulose membranes. The membranes were blocked with 5% skim milk and incubated with primary antibodies against PPAR*α*, Grp78, CHOP, Caspase12, cleaved-Caspase3, Bcl2, Bax, and *β*-actin at 4°C overnight. Then, the membranes were incubated with HRP-labeled secondary antibody for 1 h, and the protein was observed with ECL chemiluminescence kit. Quantitative analyses of immunoblotting were performed by ImageJ software.

### 2.13. RNA Extraction and Quantitative Real-time PCR

Total RNA was extracted by using TRIzol reagent and reverse transcribed into cDNA. RT-qPCR was performed using SYBR Green® kit. The expression level of mRNA was analyzed by Roche LightCycler® 96 Detection System. Relative expression values were normalized to *β*-actin control. Primer sequences are listed in Table 1.

### 2.14. PPAR*α* siRNA Transfection

The HepG2 cells were cultured in 6-well plates at an appropriate density. Lipofectamine™ 3000 (Invitrogen) was uniformly mixed with PPAR*α* siRNA and NC-siRNA (GenePharma Co., Ltd., China) in Opti-MEM and added to a 6-well plate for transfection. After 24 h incubation, the transfected cells were conducted with different treatment. Then, after drug administration and OGD/R, the cell protein was collected for further analysis.

### 2.15. Statistical Analysis

Data are represented as mean ± SD values. The *T*-test or one-way ANOVA was applied to analyze the differences between groups using SPSS 16.0. *p* < 0.05 was considered statistically significance, and *p* < 0.01 indicated a strongly significant difference.

## 3. Results

### 3.1. OEA Treatment Protects the Liver from I/R Injury

**Figure 1:**
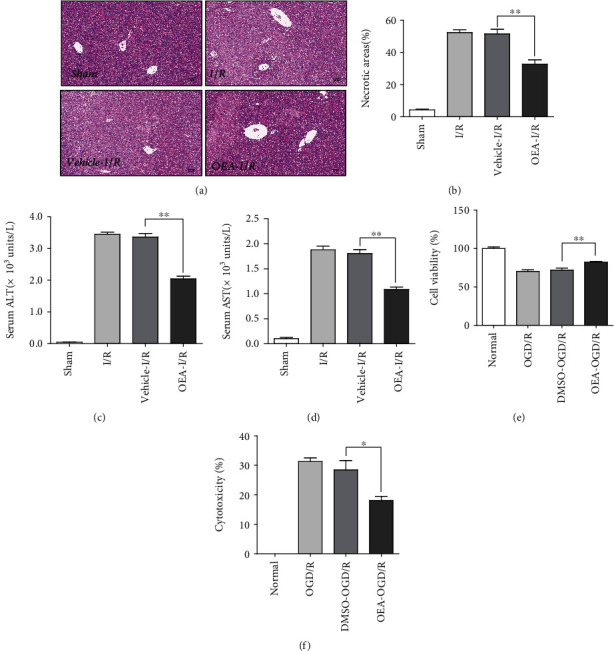
OEA treatment protects liver I/R injury. OEA treatment in mice was conducted by intraperitoneal (20 mg/kg) for 1 h before ischemia. The liver tissue and serum were harvested after reperfusion 6 h. OEA treatment of HepG2 cells (20 *μ*M) was conducted for 1 h before OGD/R. (a) Representative HE-stained images after reperfusion 6 h or sham group. (b) Percentage of necrotic areas. (c and d) The level of serum ALT and AST. (e and f) Cell viability and cytotoxicity of HepG2 cells after OGD/R with OEA or DMSO treatment were measured. ^∗∗^*p* < 0.01 and ^∗^*p* < 0.05.

To identify whether OEA has a protective effect, an *in vivo* model of partial warm ischemia-reperfusion was used to determine whether OEA prevents hepatic I/R damage in mice. It was found that OEA treatment at 20 mg/kg during liver ischemia revealed a marked decrease in the serum levels of ALT and AST (Supplementary Fig 1A and B). Histological analysis revealed that liver treated with OEA exhibited better-preserved liver tissue architecture and significantly smaller necrosis area compared to vehicle control (Figures 1(a) and 1(b)). Additionally, OEA-treated mice were revealed a significant decrease in serum ALT and AST (Figures 1(c) and 1(d)).

To detect the effect of OEA on hepatocytes, the OGD/R model of the HepG2 cells was adopted to simulate I/R injury *in vitro*. Cell CCK-8 analysis revealed that the cell viability was considerably decreased in the control than in normal group. Pretreatment with OEA (10 *μ*M) significantly suppressed OGD/R-induced death of hepatocytes (Supplementary Fig 2). Additional assays showed that OEA treatment significantly improved cell viability and reduced cytotoxicity (Figures 1(e) and 1(f)). These observations suggested that OEA treatment protected the liver from I/R injury.

### 3.2. OEA Protects against Liver I/R Injury-Induced Hepatocytes Apoptosis

**Figure 2:**
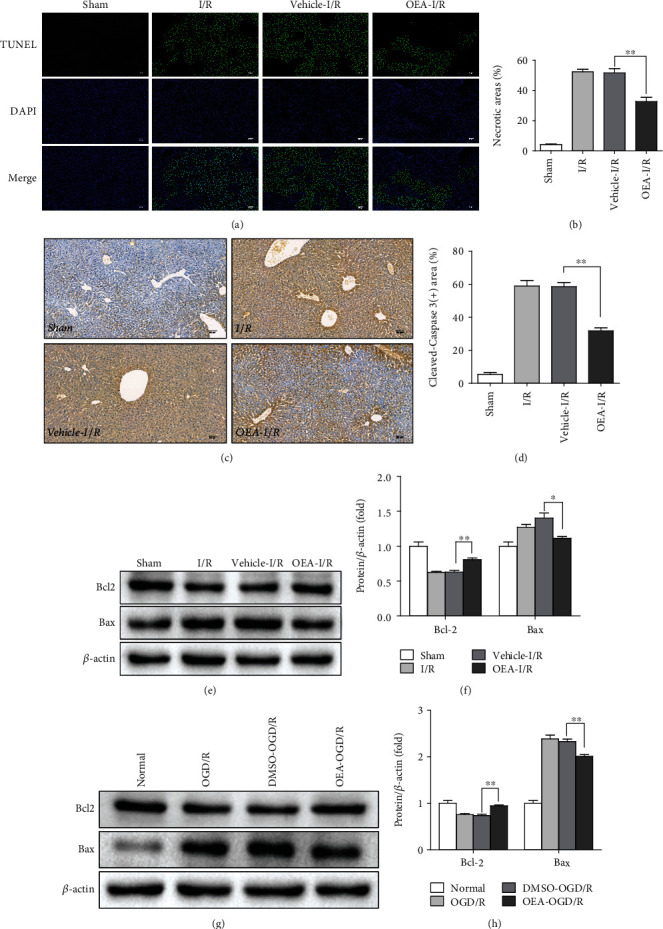
OEA pretreatment attenuates apoptosis in liver I/R injury. (a and b) TUNEL staining and the percentage of TUNEL-positive cells were, respectively, evaluated in the liver sections. (c and d) Cleaved-Caspase3 staining and the percentage of cleaved-caspase3-positive cells were, respectively, evaluated in liver sections. (e and f) Western blotting analysis of Bcl2 and Bax expression in liver tissues. (g and h) Western blotting analysis of Bcl2 and Bax expression in HepG2 cells after OGD/R. ^∗∗^*p* < 0.01 and ^∗^*p* < 0.05.

Apoptosis is an important mechanism for inducing hepatocyte death during liver I/R injury [16]. To explore the effect of OEA on liver I/R injury, TUNEL staining and immunohistochemistry staining of cleaved-Caspase3 were performed. Compared with the sham group, cleavage of Caspase3 and the percentage of TUNEL-positive cells significantly increased in the I/R group, which was attenuated by OEA treatment (Figures 2(a)–2(d)). The results of western blotting analysis further showed the downregulation of Bcl2 (antiapoptotic) and upregulation of Bax (proapoptotic) in I/R group, suggesting that I/R mediated apoptosis *in vivo*. However, the level of Bcl2 was increased, while Bax was decreased in OEA treatment group (Figures 2(e) and 2(f)). Consistently, OEA treatment produced the same effect in the OGD/R-treated HepG2 cells *in vitro* (Figures 2(g) and 2(h)). These results suggested that OEA treatment reduced hepatocyte apoptosis during liver I/R injury.

### 3.3. OEA Activates PPAR*α* to Ameliorate Cell Apoptosis

**Figure 3:**
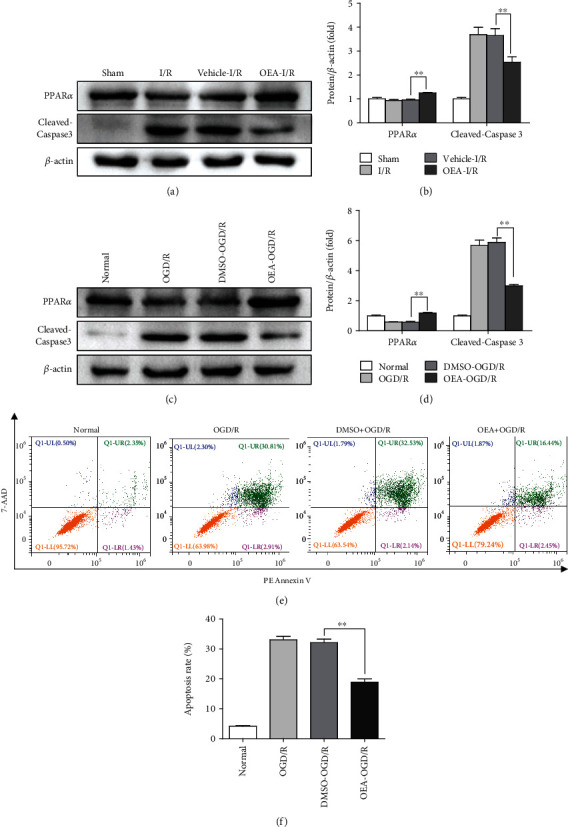
OEA activates PPAR*α* to ameliorate cell apoptosis. OEA treatment of HepG2 cells (20 *μ*M) was conducted for 1 h before OGD/R. (a and b) Western blotting analysis of PPAR*α* and cleaved-Caspase3 after liver I/R injury. (c and d) Western blotting analysis of PPAR*α* and cleaved-Caspase3 expression in OGD/R-treated HepG2 cells. (e) The apoptosis from indicated groups was detected by flow cytometry. (f) Data are based on three independent assays (*n* = 3). ^∗∗^*p* < 0.01 and ^∗^*p* < 0.05.

OEA is a high-affinity agonist of PPAR*α*. We found that the protein expression of PPAR*α* was downregulated at 6 h after reperfusion, and OEA treatment remarkably upregulated it and its target gene expression (Supplementary Fig 3). Moreover, the level of cleaved-Caspase3 was decreased significantly in the OEA-treated group compared to the control group (Figures 3(a) and 3(b)). Meanwhile, western blotting results found the same phenomena in OEA-treated HepG2 group (Figures 3(c) and 3(d)). Furthermore, flow cytometry analysis results showed that OEA treatment alleviated apoptosis in OGD/R-treated HepG2 cells (Figures 3(e) and 3(f)). These results demonstrated that OEA treatment ameliorated the apoptosis of hepatocytes during liver I/R injury.

### 3.4. OEA-Induced PPAR*α* Activation Attenuates ER Stress-Associated Apoptosis

**Figure 4:**
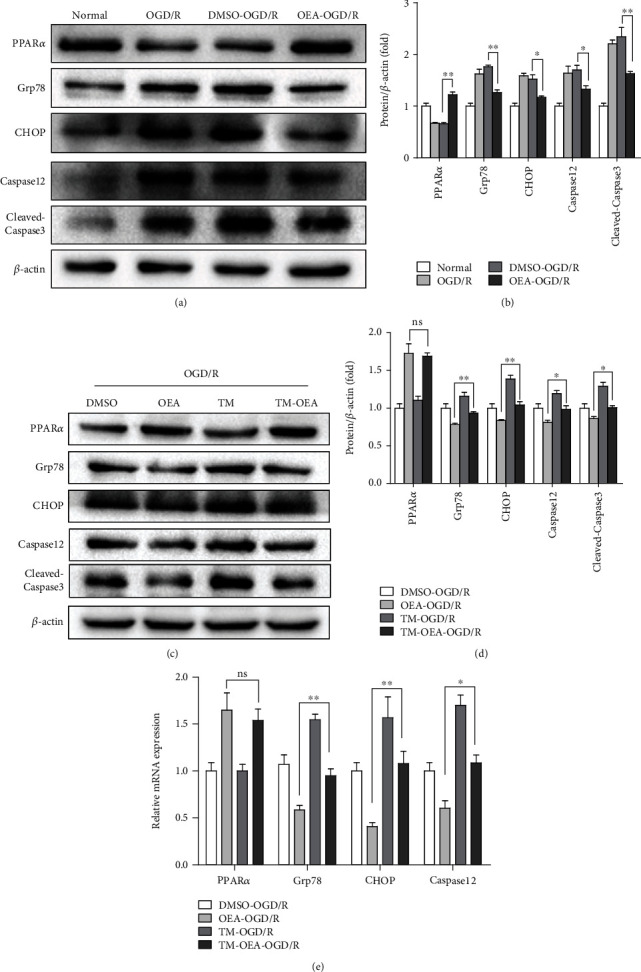
OEA induced-PPAR*α* activation attenuates ER stress-associated apoptosis. TM treatment of HepG2 cells (1 *μ*g/ml) was conducted for 6 h, and OEA treatment (20 *μ*M) was conducted for 1 h before OGD/R. The proteins were harvested at 6 h after reoxygenation. (a and b) Western blotting analysis of PPAR*α*, Grp78, CHOP, Caspase12, and cleaved-Caspase3 expression. (c and d) HepG2 cells were treated with TM for 6 h or OEA for 1 h before OGD/R operation. Western blotting analysis of PPAR*α*, Grp78, CHOP, Caspase12, and cleaved-Caspase3 expression. (e) The relative mRNA expression of PPAR*α*, Grp78, CHOP, and Caspase12. ^∗∗^*p* < 0.01 and ^∗^*p* < 0.05.

Excessive or prolonged activation of ER stress is vital in the pathogenesis of liver ischemia-reperfusion injury. ER stress triggers the activation of CHOP and Caspase12, which participate in ER-associated apoptosis. Therefore, to investigate whether OEA reduces hepatocyte apoptosis which depends on ER stress during I/R injury, the associated apoptotic markers, Grp78, CHOP, Caspase12, and cleaved-Caspase3, were analyzed. Our results confirmed that OGD/R induced ER stress-associated apoptosis as evidenced by the expression of Grp78, CHOP, Caspase12, and cleaved-Caspase3. When the cells were pretreated with OEA, the expression of those related proteins was significantly decreased (Figures 4(a) and 4(b)). TM activates UPR in mammalian cells by inhibiting N-linked glycosylation of nascent proteins, which is commonly used to induce excessive ER stress, which mediates ER stress-associated apoptosis [17–20]. To explore whether OEA protects hepatocyte against ER stress-associated apoptosis during OGD/R, the HepG2 cells were pretreated with TM before OGD/R treatment. The results showed that TM increased the expressions of Grp78, CHOP, Caspase12, and cleaved-Caspase3, while OEA treatment notably suppressed the expressions of those related proteins (Figures 4(c) and 4(d)). In addition, compared to OEA-OGD/R group, the mRNA expression of Grp78, CHOP, and Caspase12 was increased significantly in TM-OEA-OGD/R group, and the PPAR*α* mRNA expression had no difference between these two groups (Figure 4(e)). These findings indicated that OEA-induced PPAR*α* activation attenuated ER stress-associated apoptosis during OGD/R.

### 3.5. OEA Inhibits ER Stress-Associated Apoptosis to Protect Liver I/R Injury

**Figure 5:**
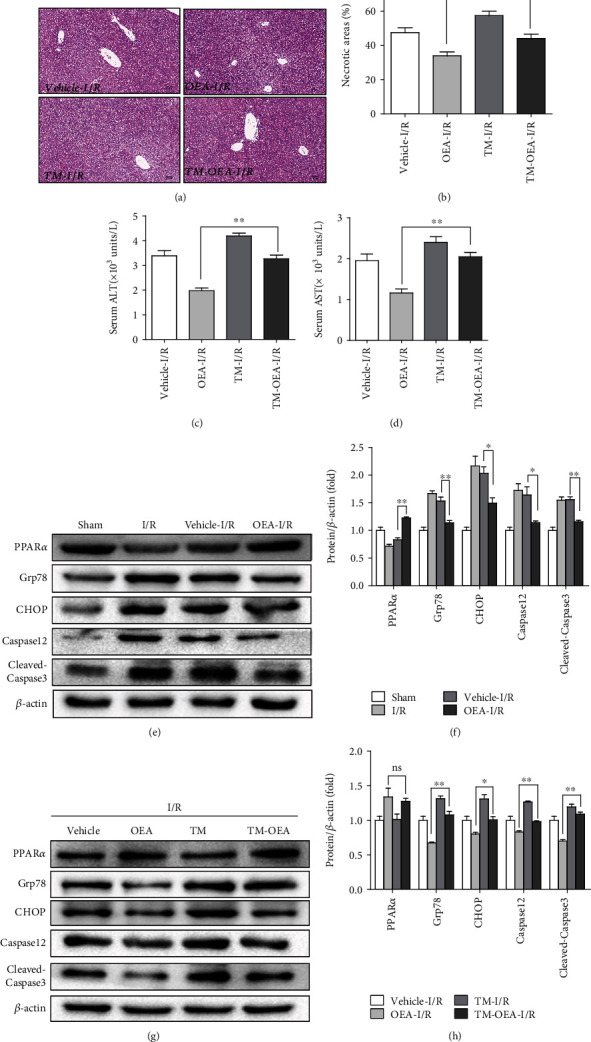
OEA inhibits ER stress-associated apoptosis to protect liver I/R injury. The mice were treated with TM by intraperitoneal (1 mg/kg) for 24 h, and OEA treatment was conducted for 1 h before the beginning of ischemia. The liver tissue and serum were harvested at 6 h after reperfusion. TM treatment of HepG2 cells (1 *μ*g/ml) was conducted for 6 h, and OEA treatment (20 *μ*M) was conducted for 1 h before OGD/R. The proteins were harvested 6 h after reoxygenation. (a and b) H&E staining and the percentage of necrotic area of the liver section. (c and d) Serum ALT/AST levels. (e and f) Western blotting analysis of PPAR*α*, Grp78, CHOP, Caspase12, and cleaved-Caspase3 expression after OEA or vehicle treatment. (g and h) TM administration for 24 h before ischemia and harvest tissue protein at 6 h after reperfusion. Western blotting analysis of PPAR*α*, Grp78, CHOP, Caspase12, and cleaved-Caspase3 expression alternation. ^∗∗^*p* < 0.01 and ^∗^*p* < 0.05.

Considering that PPAR*α* activation by OEA ameliorates ER stress-associated apoptosis, we next decided to confirm that OEA could exert hepatic I/R protection *in vivo*. OEA treatment ameliorated I/R injury significantly and TM pretreatment reversed hepatic protection induced by OEA, suggesting that at least some of the protective properties of OEA were mediated by ER stress-associated apoptosis (Figures 5(a) and 5(b)). In accordance with the histological data, compared to OEA-I/R group, the levels of ALT and AST were increased significantly in TM-OEA-I/R group (Figures 5(c) and 5(d)). Consistently, western blotting indicated that the expression of Grp78, CHOP, Caspase12, and cleaved-Caspase3 was decreased in the OEA treatment group (Figures 5(e) and 5(f)). Meanwhile, compared to OEA-I/R group, the expression of Grp78, CHOP, Caspase12, and cleaved-Caspase3 was increased significantly in the TM-OEA-I/R group (Figures 5(g) and 5(h)). These findings indicated that OEA attenuated ER stress-associated apoptosis during I/R injury via activating PPAR*α*.

### 3.6. Knockdown of PPAR*α* Exacerbates ER Stress-Associated Apoptosis and Reverses the Protect Effect of OEA

**Figure 6:**
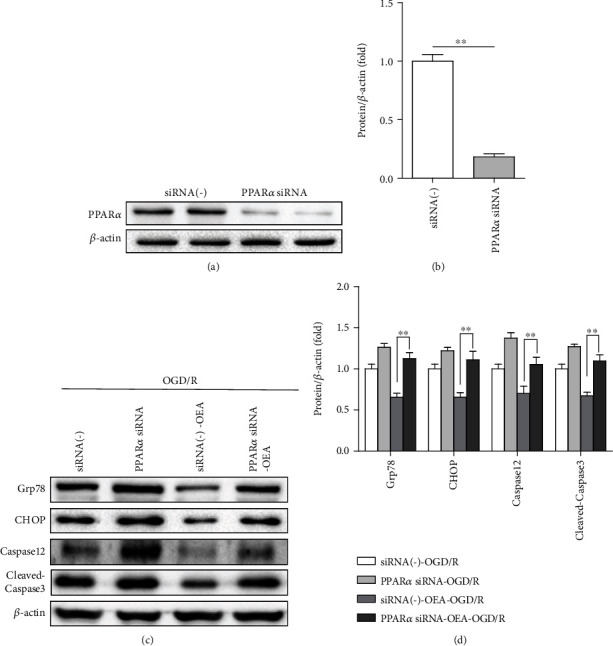
Knockdown of PPAR*α* exacerbates ER stress-associated apoptosis and reverses the protect effect of OEA in HepG2 cells. (a and b) PPAR*α* protein expression after transfected siRNA(-) or PPAR*α* siRNA. (c and d) The expression level of Grp78, CHOP, Caspase12, and cleaved-Caspase3 was measured by western blotting in HepG2 cells transfected with PPAR*α* and then treated with OEA. ^∗∗^*p* < 0.01 and ^∗^*p* < 0.05.

We used PPAR*α* siRNA to further explore whether PPAR*α* is involved in ER stress-associated apoptosis. The results showed that the expression of PPAR*α* was decreased significantly in PPAR*α* siRNA group (Figures 6(a) and 6(b)). Besides, compared to that in siRNA(-)-OEA-OGD/R group, the protein expression of Grp78, CHOP, Caspase12, and cleaved-Caspase3 was significantly increased in the PPAR*α* siRNA-OEA-OGD/R group (Figures 6(c) and 6(d)). Therefore, all these observations confirmed that OEA protected the liver against hepatic I/R injury, at least in part, via activating PPAR*α* to inhibit ER stress-associated apoptosis.

## 4. Discussion

**Figure 7:**
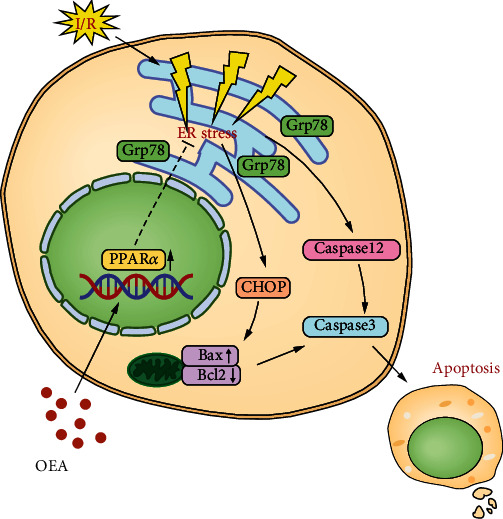
Mechanism of OEA alleviating liver I/R injury. OEA upregulates PPAR*α* expression, promotes the nuclear translation of PPAR*α*, inhibits ER stress-associated apoptosis, and alleviates liver I/R injury.

The present study demonstrated that OEA protects liver I/R injury *in vivo* and *in vitro*. The results showed that OEA ameliorates liver I/R injury, indicating that OEA has a potent protective role by activating PPAR*α*. Mechanistically, we found that OEA inhibits ER stress-associated apoptosis (Figure 7), which may provide clues for new therapeutic targets of liver I/R injury.

Liver I/R injury is a severe complication that occurs after circulatory shock and liver transplantation, leading to high mortality and morbidity. OEA is an endogenous ligand of PPAR*α*, and it plays protective roles on brain I/R injury [21–23]. However, there are no reports about the function of OEA in liver I/R injury. In our study, our data demonstrated that OEA alleviates hepatic ischemia-reperfusion injury.

Increasing evidence showed that ER stress-associated apoptosis plays an vital role in liver I/R injury. ER stress mediates apoptosis through three main pathways: activation pathways of Caspase12 and JNK and transcription of the CHOP/GADD153 gene [24]. Specifically, Grp78 is separated from the receptors when ER stress is activated and repaired normal function of ER by activating related signaling pathways to improve protein folding [25, 26]. CHOP is a crucial transcription factor facilitating ER stress-mediated apoptosis. Under normal circumstances, CHOP maintained a low level, while excessive ER stress increased the expression of CHOP, eventually generating cell apoptosis [27]. CHOP downregulates Bcl2 transcription and upregulates Bax expression, connecting ER stress with mitochondrial apoptosis [28]. Caspase12 is known as the initiator and key factor involved in ER stress-associated apoptosis [29]. Here, we found that OEA decreases ER stress-associated apoptosis during liver I/R injury. Therefore, inhibition of excessive ER stress response may be an ideal strategy for preventing or intervening hepatic I/R injury.

We found PPAR*α* was significantly inhibited in hepatic I/R injury. OEA dramatically increased the expression level of PPAR*α* and decreased apoptosis of hepatocytes, indicating an important role for PPAR*α* in the hepatoprotection. A previous study has revealed that PPAR*α* activation decreased severe ER stress-induced hepatocyte apoptosis in acute liver failure [30]. In this study, it was found that I/R injury or OGD/R could significantly aggravate ER stress-associated apoptosis. The expression levels of CHOP, Caspase12, and cleaved-Caspase3 were significantly higher than those in control group. In addition, pretreatment with OEA can alleviate ER stress-associated apoptosis, as evidenced by the decrease in those related proteins both *in vivo* and *in vitro*. Besides, knockdown of PPAR*α* increased ER stress-associated apoptosis and abolished the protect effect of OEA. Therefore, PPAR*α* signaling is important in hepatoprotection and suggests that the modulation of OEA may protect against clinical liver I/R injury.

Taken together, our data indicated that PPAR*α* signaling pathway was critical for the pathogenesis of hepatic I/R. OEA-mediated PPAR*α* activation attenuates liver I/R injury, at least partly through inhibiting ER stress-associated apoptosis. Moreover, ER stress is perceived as an attractive potential target, which may ameliorate pathological process of liver I/R via inhibiting ER stress. OEA may serve as a potential hepatoprotective agent in the clinical setting of ischemia and organ preservation.

## Figures and Tables

**Table 1:** Primer sequences for qRT-PCR.

Gene	Primer pair	
PPAR*α*	F:5′-ATGGTGGACACGGAAAGCC-3′	R:5′-CGATGGATTGCGAAATCTCTTGG-3′
Grp78	F:5′-CATCACGCCGTCCTATGTCG-3′	R:5′-CGTCAAAGACCGTGTTCTCG-3′
CHOP	F:5′-GGAAACAGAGTGGTCATTCCC-3′	R:5′-CTGCTTGAGCCGTTCATTCTC-3′
Caspase12	F:5′-AACAACCGTAACTGCCAGAGT-3′	R:5′-CTGCACCGGCTTTTCCACT-3′
GADPH	F:5′-GGAGCGAGATCCCTCCAAAAT-3′	R:5′-GGCTGTTGTCATACTTCTCATGG-3′

## Data Availability

The datasets generated/analyzed during the current study are available.

## Supplementary Materials

Supplementary Materials Supplementary Materials 1: optimum concentration of OEA *in vivo*. We compared sham group, vehicle group, and OEA groups conducted different concentration and detected serum ALT and AST levels of those groups. The results were exhibited as follows. We found that 20 mg/kg was the optimum concentration of OEA *in vivo*. Supplementary Materials 2: optimum concentration of OEA *in vitro*. We detected cell viability by measuring the absorbance at 450 nm to determine the optimum concentration of OEA. We found that 10 *μ*M was the optimum concentration of OEA *in vitro*. Supplementary Materials 3: the primer sequences of PPAR*α* target genes for qRT-PCR and the mRNA expression level of PPAR*α* target genes.
