# Supplementary material for: Oleoylethanolamide Alleviates Hepatic Ischemia-Reperfusion Injury via Inhibiting Endoplasmic Reticulum Stress-Associated Apoptosis
Source: PPAR Res. 2022 Mar 21;2022:2212996. doi: 10.1155/2022/2212996 (PMC8960015; doi:10.1155/2022/2212996)

Supplementary Figure 1

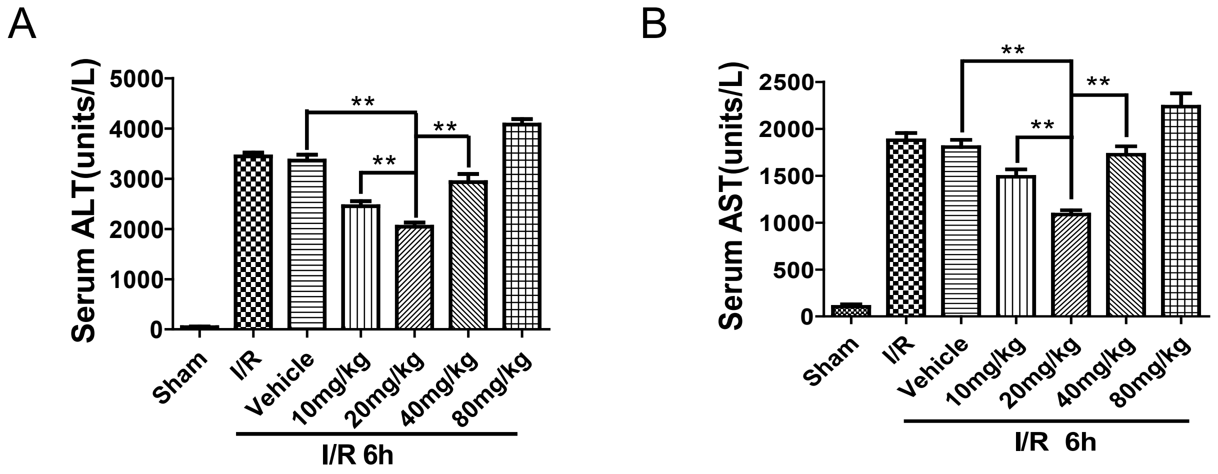

Supplementary Figure 2

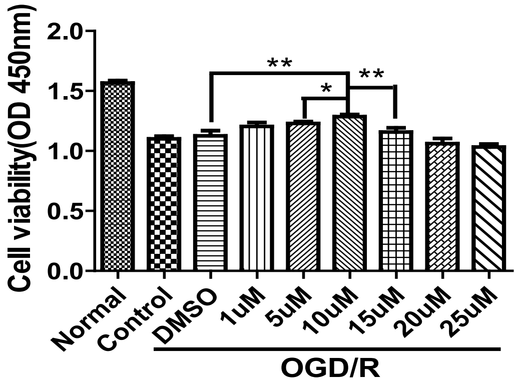

Supplementary Figure 3

Primer sequences for qPCR

Gene	Primer pair	
Cpt1a	F:5'- TCCAGTTGGCTTATCGTGGTG-3’	R:5'- TCCAGAGTCCGATTGATTTTTGC-3’
Acox	F:5'- ACTCGCAGCCAGCGTTATG-3’	R:5'- AGGGTCAGCGATGCCAAAC-3’
Fatp2	F:5'- TTTCCGCCATCTACACAGTCC-3’	R:5'- CGTAGGTGAGAGTCTCGTCG-3’
GADPH	F:5'- GGAGCGAGATCCCTCCAAAAT-3’	R:5'- GGCTGTTGTCATACTTCTCATGG-3’

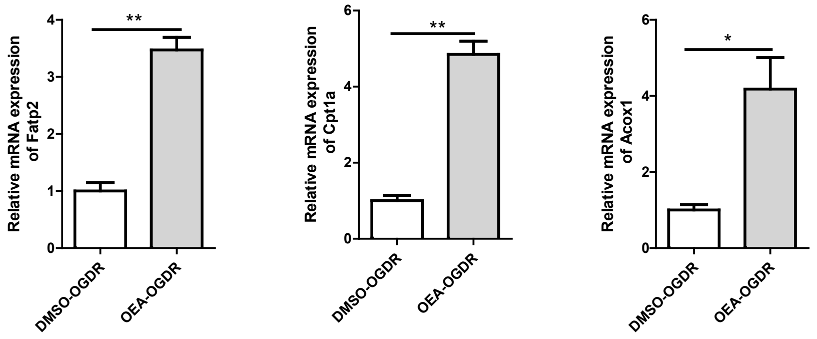

Supplement: Supplementary Materials — 1: optimum concentration of OEA in vivo. We compared sham group, vehicle group, and OEA groups conducted different concentration and detected serum ALT and AST levels of those groups. The results were exhibited as follows. We found that 20 mg/kg was the optimum concentration of OEA in vivo. Supplementary Materials 2: optimum concentration of OEA in vitro. We detected cell viability by measuring the absorbance at 450 nm to determine the optimum concentration of OEA. We found that 10 μM was the optimum concentration of OEA in vitro. Supplementary Materials 3: the primer sequences of PPARα target genes for qRT-PCR and the mRNA expression level of PPARα target genes. [file 2212996.f1.pdf]
